# Effect of transient obstructive cholestasis on liver histology: a cross-sectional study

**DOI:** 10.1590/1516-3180.2020.0536.R1.1502021

**Published:** 2021-06-11

**Authors:** Thalita Mendes Mitsunaga, Laísa Simakawa Jimenez, Pedro França da Costa Soares, Martinho Antonio Gestic, Murillo Pimentel Utrini, Felipe David Mendonça Chaim, Francisco Callejas-Neto, Elinton Adami Chaim, Everton Cazzo

**Affiliations:** I MD. Resident Physician, Department of Surgery, Faculdade de Ciências Médicas da Universidade de Campinas (FCM-UNICAMP), Campinas (SP), Brazil.; II MD. Resident Physician, Department of Surgery, Faculdade de Ciências Médicas da Universidade de Campinas (FCM-UNICAMP), Campinas (SP), Brazil.; III MD, MSc. Postgraduate Student, Department of Surgery, Faculdade de Ciências Médicas da Universidade de Campinas (FCM-UNICAMP), Campinas (SP), Brazil.; IV MD, MSc. Attending Physician, Department of Surgery, Faculdade de Ciências Médicas da Universidade de Campinas (FCM-UNICAMP), Campinas (SP), Brazil.; V MD. Attending Physician, Department of Surgery, Faculdade de Ciências Médicas da Universidade de Campinas (FCM-UNICAMP), Campinas (SP), Brazil.; VI MD, PhD. Attending Physician, Department of Surgery, Faculdade de Ciências Médicas da Universidade de Campinas (FCM-UNICAMP), Campinas (SP), Brazil.; VII MD, MSc. Assistant Professor, Department of Surgery, Faculdade de Ciências Médicas da Universidade de Campinas (FCM-UNICAMP), Campinas (SP), Brazil.; VIII MD, PhD. Full Professor, Department of Surgery, Faculdade de Ciências Médicas da Universidade de Campinas (FCM-UNICAMP), Campinas (SP), Brazil.; IX MD, PhD. Adjunct Professor, Department of Surgery, Faculdade de Ciências Médicas da Universidade de Campinas (FCM-UNICAMP), Campinas (SP), Brazil.

## Abstract

**BACKGROUND:**

The role of transient obstructive cholestasis on liver histology remains undetermined.

**OBJECTIVE:**

To investigate whether transient cholestasis impairs liver histology.

**DESIGN AND SETTING:**

Cross-sectional study at a public university hospital (UNICAMP), Brazil.

**METHODS:**

169 individuals undergoing cholecystectomy, with or without cholestasis. were enrolled. Histopathological findings were correlated with clinical and biochemical characteristics.

**RESULTS:**

Biliary hepatopathy was more frequent in individuals with resolved cholestasis than in those with active obstruction or no jaundice (P < 0.01), as also were fibrosis and ductular proliferation (P = 0.02). Cholestasis was commoner in individuals with resolved obstruction than in those with no history (P < 0.01) or active cholestasis (P < 0.05). Biliary hepatopathy was associated with longer duration of cholestasis (P < 0.001) and higher bilirubin levels (P = 0.02) in individuals with active obstruction; with lower body mass index (P = 0.02) and longer cholestasis (P < 0.001) in individuals with resolved obstruction; and with longer cholestasis (P < 0.001) and longer interval between endoscopic retrograde cholangiopancreatography and surgery (P = 0.03) overall. In individuals with active obstruction, duration of cholestasis (R = 0.7; P < 0.001) and bilirubin levels (R = 0.6; P = 0.004) were independently correlated with cholestasis severity. Duration of cholestasis (R = 0.7; P < 0.001) was independently correlated with ductular proliferation severity.

**CONCLUSIONS:**

Transient cholestasis was associated with significant histopathological changes, even after its resolution. Longer duration of obstruction correlated with greater severity of histopathological changes, especially cholestasis and ductular proliferation. This emphasizes the need for early treatment of obstructive cholestasis.

## INTRODUCTION

Cholestasis is an impairment of bile formation and/or bile flow that may present with fatigue, pruritus and, in its most suggestive form, jaundice. It can be classified as intrahepatic or extra-hepatic.^1^^,^^2^^,^^3^^,^^4^ It may assume various histological patterns, which present different clinical and diagnostic connotations. The two main patterns, which are canalicular cholestasis and chronic cholestasis, constitute general categories, more suggestive of the progress and degree of cholestasis than of any exact cause. The other two patterns, ductular cholestasis and ductal cholestasis, usually develop within a context of canalicular cholestasis, but are differentiated because of their narrower clinical contexts. Basically, the main histological features observed in cholestasis are occurrences of ductular proliferation and bilirubinostasis.^5^ Biliary obstruction is caused by mechanical impairment of bile flow through large ducts, mainly extrahepatic bile ducts. Its structural correspondent is the parenchymal cholestasis with biliary stasis located in zone 3 (perivenular). In cases of incomplete obstruction, a ductular reaction may occur without clear evidence of cholestasis.^6^

Several studies have shown severe consequences of biliary obstruction on liver histology, with changes such as microscopic cholestasis and cholangitis, liver fibrosis and inflammatory changes. Prolonged maintenance may even lead to biliary cirrhosis.^5^^,^^6^^,^^7^ The vast majority of cases of biliary obstruction are transient, since several treatments can usually be employed to correct the obstructive factor. Treatment may, in most cases, be either surgical or endoscopic. There are cases in which the obstruction resolves spontaneously, such as when small gallstones migrate through the duodenal papilla after a period of obstruction. Commonly, the signs and symptoms of cholestasis cease gradually after the obstructive factor resolves. However, there is no conclusive evidence regarding the role of transient cholestasis and the duration of obstruction on liver histology.^4^^,^^5^^,^^6^^,^^7^

## OBJECTIVE

The aims of this study were to investigate whether occurrence of transient cholestasis might lead to significant changes in liver histology and to analyze the influence of the duration of cholestasis on liver histology.

## METHODS

### Study design

This was a cross-sectional study that enrolled individuals who underwent cholecystectomy at a public tertiary-level university hospital between July 2018 and October 2019. The study protocol was evaluated and approved by the local institutional review board under the reference no. 3.279.991/UNICAMP (CAAE: 10628119.4.0000.5404; date: April 24, 2019). All participants provided informed consent. Liver wedge biopsies were performed on all participants during surgery (cholecystectomy), and samples were collected from hepatic segment III. The findings from histopathological examinations were correlated with the participants’ clinical and biochemical characteristics.

### Study population

This study included individuals aged 18 years or above, of both genders, who underwent cholecystectomy due to gallbladder disease. The exclusion criteria were the following: past or current history of unrelated biliary liver disease; previous unrelated intervention on the liver or biliary tree; belonging to vulnerable groups; positive serological tests for viral liver disease; past or current use of alcohol or illicit drugs; current or recent use of hepatotoxic drugs or drugs associated with cholestasis; active malignant neoplasm; or incomplete medical records.

The minimum sample size was estimated as 162 individuals, considering an alpha of 0.05, a prevalence proportion of 0.3 and precision of 10%. Out of an initial population of 185 individuals undergoing cholecystectomy, 16 participants were excluded. The causes of exclusion were the following: viral hepatitis (n = 2), liver cirrhosis (n = 3), primary sclerosing cholangitis (n = 1), alcohol use (n = 5), use of hepatotoxic drugs (n = 3) and incomplete medical records (n = 2). Thus, 169 individuals remained included in the study.

### Classification into subgroups

**Figure 1:**
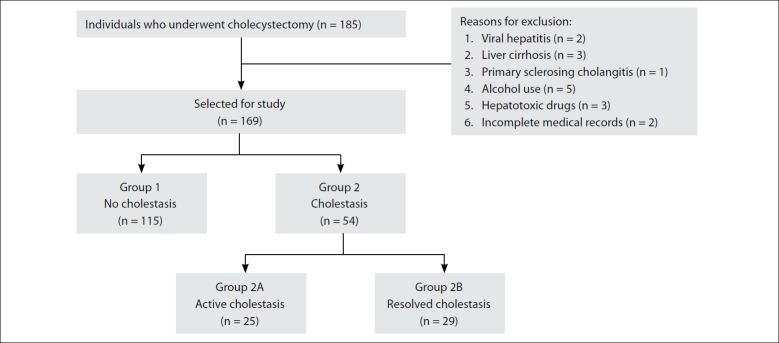
Flowchart of the study population.

The participants were classified according to their clinical histories, laboratory tests and imaging examinations into two large groups: group 1, no clinical cholestasis; and group 2, with a history of clinical cholestasis. Group 2 was subdivided into two other groups: 2A) active cholestasis (clinical history of cholestasis and/or imaging examinations with evidence of obstruction at a date close to surgery and/or ineffective endoscopic retrograde cholangiopancreatography (ERCP) and/or total bilirubin ≥ 2.5 mg/dl); and 2B) resolved cholestasis (clinical history of spontaneously resolved cholestasis and/or imaging examinations with no evidence of obstruction at a date close to surgery and/or effective ERCP and/or total bilirubin < 2.5 mg/dl). The imaging examinations considered in the evaluations included ultrasound scan, computed tomography, endoscopic cholangiography and/or magnetic resonance cholangiography. The participants were then accordingly divided between these groups, as follows: 1 (n = 115); 2 (n = 54); 2A (n = 25); and 2B (n = 29). Figure 1 shows the flowchart of the study population and the subdivision according to groups.

Additionally, individuals were divided into groups according to their bilirubin levels (< 3 mg/dl, 3-7 mg/dl or ≥ 7 mg/dl). Those who presented cholestasis were also divided into two groups according to the duration of obstruction (< 15 days or ≥ 15 days).

### Variables

The following clinical and demographic variables were analyzed: age (in years); gender (male or female); cholestasis status (active, resolved, overall or no history of cholestasis); endoscopic retrograde cholangiopancreatography (performed or not performed); duration of cholestasis (estimated in days from clinical history information in the medical records); interval between ERCP and surgery (in days); and body mass index (BMI; expressed in kg/m^2^).

The following biochemical variables were considered: total bilirubin (in mg/dl); aspartate aminotransferase (AST; in mg/dl); alanine aminotransferase (ALT; in mg/dl); alkaline phosphatase (ALP; in mg/dl); gamma-glutamyl transferase (GGT; in mg/dl); albumin (ALB; in g/dl); and international normalized ratio (INR).

The histopathological variables considered were the present or absence of biliary pattern liver disease and the following specific histological characteristics: fibrosis, ductular proliferation, cholestasis, portal inflammation, steatosis and cholangitis. These characteristics were classified dichotomously as absent or present and were also stratified ordinally according to their degree of severity, as absent (0), mild (1), moderate (2) or severe (3).^5^ The histopathological analyses were all performed by the same pathology team and followed the same parameters as defined by Crawford et al.^8^

All specimens were fixed in formalin, embedded in paraffin and sectioned using a microtome at a thickness of 5 μm. Routine specimen processing involved staining the slides with hematoxylin and eosin (15 levels), Masson trichrome (10 levels) and reticulin (5 levels), for a total of 30 levels per specimen. All levels were screened to ensure absence of histological abnormalities.^8^

### Statistical analysis

The descriptive analysis consisted of presenting frequency tables for categorical variables and dispersion measurements for numerical variables. For comparison of proportions, we used the chi-square test or Fisher’s exact test, when necessary. For comparison of continuous variables between two groups, the Mann-Whitney test was used; for comparison between three or more, the Kruskal-Wallis test was used, with application of the Tukey post-test. For univariate analysis on associations between ordinal endpoints and continuous variables, simple linear regression models were used; for multivariate analysis on significant associations, multiple regression models were used. The significance level used for the statistical tests was 5% (P < 0.05). To perform the analyses, the SAS System for Windows computer software (Statistical Analysis System), version 9.2, was used (SAS Institute Inc., 2002-2008; Cary, NC, United States).

## RESULTS

### Demographic characteristics and nutritional status

There was a predominance of females in the study population (65.7%). There were no differences in gender distribution between the groups (P = 0.9). The average age was 47.2 ± 17.3 years; there were no significant differences in age between the groups studied (P = 0.6). The mean BMI was 27.4 ± 5.2 kg/m^2^; there were no significant differences between the groups (P = 0.08). The complete comparison among the groups is presented in **Table 1**.

**Table 1:** Demographic characteristics, nutritional status and biochemical variables of the study population

**N**	**No cholestasis (group 1)**	**Active jaundice (group 2A)**	**Resolved cholestasis (group 2B)**	**P-value (Post-test P-value when applicable)**

	**115**	**25**	**29**	**NA**
Age (years)	46.9 ± 17.9	45.4 ± 16.9	50.1 ± 19.8	0.6
Gender	F: 77 (67%)	F: 16 (64%)	F: 18 (62.1%)	0.9
	M: 38 (33%)	M: 9 (36%)	M: 11 (37.9%)	
BMI (kg/m^2^)	27.9 ± 5.1	27.1 ± 5	25.4 ± 5	0.08
Bilirubin (mg/dl)	0.6 ± 0.2	5.5 ± 4.5	1 ± 0.5	*< 0.001* *(2A > 2B; P < 0.01)* *(2A > 1; P < 0.01)*
Aspartate aminotransferase (mg/dl)	19.7 ± 7.6	225 ± 210.3	30.1 ± 16.8	*< 0.001* *(2A > 2B; P < 0.01)* *(2A > 1; P < 0.01)*
Alanine aminotransferase (mg/dl)	21.2 ± 13.3	450.1 ± 722.2	46.2 ± 37.6	*< 0.001* *(2A > 2B; P < 0.01)* *(2A > 1; P < 0.01)*
Alkaline phosphatase (mg/dl)	71 ± 25.1	327 ± 295.5	95.3 ± 59.3	*< 0.001* *(2A > 1; P < 0.01)* *(2A > 2B; P < 0.01)*
Gamma-glutamyl transferase (mg/dl)	39.8 ± 35	457.3 ± 380.1	123.6 ± 158	*< 0.001* *(2A > 2B; P < 0.01)* *(2A > 1; P < 0.01)*
Albumin (g/dl)	4.2 ± 0.3	4 ± 0.6	4.2 ± 0.4	*0.01* *(2A < 2B; P < 0.05)*
International normalized ratio	1 ± 0.1	1.3 ± 0.2	1.2 ± 0.1	*< 0.001* *(2A > 1; P < 0.01)* *(2B > 1; P < 0.01)*

N = number of individuals; BMI = body mass index; F = female; M = male; NA = not applicable. Italics indicate statistical significance.

### Biochemical variables

Regarding biochemical variables, group 2A showed significantly higher levels of bilirubin (P < 0.001), AST (P < 0.001), ALT (P < 0.001), ALP (P < 0.001) and GGT (P < 0.001), compared with groups 1 and 2B. Mean INR was lower in group 1 than in groups 2A and 2B (P < 0.001) and ALB was lower in group 2A than in groups 1 and 2B (P = 0.01). The complete comparison among biochemical variables is presented in **Table 1**.

### Distribution of histopathological variables

It was observed that the presence of biliary hepatopathy was more frequent in group 2B than in groups 1 and 2A (P < 0.01) and also in group 2A compared with group 2 (P < 0.05). Fibrosis was more common in group 2B than in groups 1 and 2A (P = 0.02), as also was ductular proliferation (P = 0.02). Cholestasis was more common in group 2B than in groups 2A (P < 0.01) and 1 (P < 0.05) and was also more frequent in group 2A than in group 1 (P < 0.05). There were no significant differences in the distribution of portal inflammation (P = 1.0), cholangitis (P = 0.6) or steatosis (P = 0.3). The complete distribution of histopathological variables is presented in **Table 2**.

**Table 2:** Distribution of histopathological variables according to subgroup classification

	**No cholestasis (group 1)**	**Active cholestasis (group 2A)**	**Resolved cholestasis (group 2B)**	**Value of P (Post-test value of P when applicable)**
Biliary hepatopathy N (%)	25 (21.7%)	11 (44%)	24 (82.8%)	*< 0.001* *(2B > 1; P < 0.01)* *(2B > 2A; P < 0.01)* *(2A > 1; P < 0.05)*
Fibrosis N (%)	63 (54.8%)	13 (52%)	24 (82.8%)	*0.02* *(2B > 1; P < 0.05)* *(2B > 2A; P < 0.05)*
Ductular proliferation N (%)	39 (33.9%)	9 (36%)	18 (62.1%)	*0.02* *(2B > 1; P < 0.05)* *(2B > 2A; P < 0.05)*
Cholestasis N (%)	25 (21.7%)	9 (36%)	16 (55.2%)	*0.001* *(2B > 1; P < 0.01)* *(2B > 2A; P < 0.05)* *(2A > 1; P < 0.05)*
Portal inflammation N (%)	37 (32.2%)	8 (32%)	10 (34.5%)	1.0
Steatosis N (%)	50 (43.5%)	9 (36%)	8 (27.6%)	0.3
Cholangitis N (%)	0	2 (8%)	0	0.6

N = number of individuals. Italics indicate statistical significance.

### Biliary hepatopathy

In the analysis on the presence of biliary hepatopathy, it was observed that, in the overall population, its presence was associated with lower BMI (P = 0.01) and higher bilirubin levels (P = 0.01) and INR (P = 0.04). In a subgroup analysis, biliary hepatopathy was associated with longer duration of cholestasis (P < 0.001), higher bilirubin levels (P = 0.02) and higher INR (P = 0.02) in group 2A; with lower BMI (P = 0.02) and longer duration of cholestasis (P < 0.001) in group 2B; and with longer duration of cholestasis (P < 0.001) and longer interval between ERCP and surgery (P = 0.03), along with higher levels of ALP (P = 0.01) and GGT (P = 0.01) in group 2. In group 1, there was no variable that differed between the groups with or without biliary hepatopathy. The complete comparison between the variables analyzed according to the presence of biliary hepatopathy is presented in **Table 3**.

**Table 3:** Correlation between occurrence of biliary hepatopathy and variables analyzed in the study population, according to subgroup classification

***No cholestasis (group 1)***			

	**Present**	**Absent**	**P-value**
N (%)	25	90	NA
Age (years)	46.6 ± 18	48 ± 17.7	0.7
Gender	M: 6	M: 32	0.3
	F: 19	F: 58	
BMI (kg/m^2^)	23.6 ± 4.6	28.3 ± 5.1	0.1
Bilirubin (mg/dl)	0.5 ± 0.2	0.6 ± 0.3	0.1
Aspartate aminotransferase (mg/dl)	19.6 ± 9	19.7 ± 7	1.0
Alanine aminotransferase (mg/dl)	18.5 ± 9.9	21.9 ± 14.1	0.3
Alkaline phosphatase (mg/dl)	64.1 ± 19.9	72.9 ± 26.2	0.1
Gamma-glutamyl transferase (mg/dl)	28.2 ± 20.4	42.7 ± 37.7	0.07
International normalized ratio	1 ± 0.1	1.1 ± 0.1	0.5
Albumin (g/dl)	4.1 ± 0.3	4.3 ± 0.4	0.1

***Overall cholestasis (group 2)***			

	**Present**	**Absent**	**P-value**
N (%)	35	19	NA
Age (years)	47.7 ± 18	48.4 ± 20	0.9
Gender	M: 15	M: 5	0.2
	F: 20	F: 14	
BMI (kg/m^2^)	25.8 ± 5.2	27.1 ± 5.5	0.4
Duration of cholestasis (days)	37.3 ± 12.3	9.9 ± 3	< *0.001*
ERCP-surgery interval (days) (N = 17)	8.4 ± 2.5	43.7 ± 16.4	*0.03*
Bilirubin (mg/dl)	3.1 ± 4.6	3 ± 1.9	0.9
Aspartate aminotransferase (mg/dl)	93.4 ± 39.1	173.4 ± 39.7	0.1
Alanine aminotransferase (mg/dl)	214.7 ± 113.1	264.2 ± 59.4	0.8
Alkaline phosphatase (mg/dl)	312.5 ± 76.2	144.3 ± 22.5	*0.01*
Gamma-glutamyl transferase (mg/dl)	412.1 ± 96.6	180.9 ± 41.1	*0.01*
International normalized ratio	1.1 ± 0.2	1.1 ± 0.2	0.1
Albumin (g/dl)	4 ± 0.4	4.1 ± 0.6	0.5

***Active cholestasis (group 2A)***			

	**Present**	**Absent**	**P-value**
N (%)	11	14	NA
Age (years)	43.8 ± 15.3	46.7 ± 18.5	0.7
Gender	M: 5	M: 4	0.4
	F: 6	F: 10	
BMI (kg/m^2^)	28.6 ± 6.9	26 ± 4.1	0.3
Duration of cholestasis (days)	30 ± 10.5	10.4 ± 2.8	*< 0.001*
ERCP-surgery interval (days) (N = 17)	9.6 ± 10.9	22.7 ± 9.5	0.2
Bilirubin (mg/dl)	7.8 ± 5.9	3.7 ± 1.7	0.02
Aspartate aminotransferase (mg/dl)	222.6 ± 259.4	226.8 ± 172.7	1.0
Alanine aminotransferase (mg/dl)	582.5 ± 1064.4	346.1 ± 254.8	0.4
Alkaline phosphatase (mg/dl)	399.5 ± 348.5	234.6 ± 185.8	0.2
Gamma-glutamyl transferase (mg/dl)	537.6 ± 393.5	355.2 ± 393.5	0.2
International normalized ratio	1.2 ± 0.2	1 ± 0.1	*0.02*
Albumin (g/dl)	3.8 ± 0.5	4.1 ± 0.7	0.4

***Resolved cholestasis (group 2B)***			

	**Present**	**Absent**	**P-value**
N (%)	24	5	NA
Age (years)	49.5 ± 19.1	53 ± 25.4	0.7
Gender	M: 10	M: 1	0.4
	F: 14	F: 4	
BMI (kg/m^2^)	24.4 ± 3.5	30.2 ± 8.2	*0.02*
Duration of cholestasis (days)	40.6 ± 12.1	8.6 ± 3.6	< *0.001*
ERCP-surgery interval (days) (N = 17)	23.4 ± 39	70 ± 47.9	0.2
Bilirubin (mg/dl)	0.9 ± 0.5	1 ± 0.3	0.7
Aspartate aminotransferase (mg/dl)	31.5 ± 17.7	23.8 ± 10.7	0.4
Alanine aminotransferase (mg/dl)	44.2 ± 38.4	35 ± 36.5	0.6
Alkaline phosphatase (mg/dl)	101 ± 64.2	68.8 ± 4.1	0.3
Gamma-glutamyl transferase (mg/dl)	134.1 ± 172.5	72.8 ± 63.9	0.4
International normalized ratio	1.1 ± 0.1	1.1 ± 0.1	0.7
Albumin (g/dl)	4.1 ± 0.4	4.3 ± 0.3	0.1

***Overall study population (groups 1 + 2)***			

	Present	Absent	P-value
N (%)	60	109	NA
Age (years)	47.8 ± 17.7	46.9 ± 18.3	0.7
Gender	M: 21	M: 37	0.9
	F: 39	F: 72	
BMI (kg/m^2^)	26 ± 4.9	28.1 ± 5.2	*0.01*
Bilirubin (mg/dl)	2 ± 3.7	1 ± 1.2	*0.01*
Aspartate aminotransferase (mg/dl)	61.4 ± 133.6	46.4 ± 96.1	0.4
Alanine aminotransferase (mg/dl)	64.2 ± 140.9	129.1 ± 496.7	0.2
Alkaline phosphatase (mg/dl)	109.4 ± 108.3	114.7 ± 165.2	0.8
Gamma-glutamyl transferase (mg/dl)	121.3 ± 198.8	103.8 ± 216.9	0.6
International normalized ratio	1.2 ± 0.1	1 ± 0.1	*0.04*
Albumin (g/dl)	3.9 ± 0.8	4.1 ± 0.9	0.3

N = number of individuals; NA = not applicable; M = male; F = female; BMI = body mass index. Italics indicate statistical significance.

### Subgroup regression analysis

Linear regression analyses were performed between clinical variables in relation to the severity of histopathological characteristics (ordinal outcomes) in the analysis groups and in the overall study population.

In group 1, associations were observed between cholestasis severity and BMI (R = -0.3; P = 0.01) and ALB levels (R = -0.3; P = 0.01) and between severity of ductular proliferation and BMI (R = -0.3; P = 0.005) and ALB levels (R = -0.2; P = 0.04). After multiple regression, it was observed that both ALB (R = -0.2; P = 0.02) and BMI (R = -0.2; P = 0.02) were independently correlated with cholestasis severity and that BMI (R = -0.2; P = 0.008) was independently correlated with the severity of ductular proliferation.

In group 2, significant associations were observed between fibrosis severity and ALP levels (R = 0.4; P = 0.002) and between ductular proliferation severity and duration of cholestasis (R = 0.6; P < 0.001).

In group 2A, significant correlations were observed between fibrosis severity and ALP (R = 0.4; P = 0.03) and GGT levels (R = 0.5; P = 0.01); between cholestasis severity and duration of cholestasis (R = 0.8; P < 0.001), bilirubin levels (R = 0.6; P = 0.004), INR (R = 0.3; P = 0.04) and ALB (R = -0.3; P < 0.001); and between ductular proliferation severity and duration of cholestasis (R = 0.7; P < 0.001), INR (R = 0.7; P = 0.03) and ALB (R= -0.7; P < 0.001).

After multiple regression, GGT was independently correlated with fibrosis severity (R = 0.5; P = 0.006); duration of cholestasis (R = 0.7; P < 0.001) and bilirubin levels (R = 0.6; P = 0.004) were independently correlated with cholestasis severity; and duration of cholestasis (R = 0.7; P < 0.001) was independently correlated with the severity of ductular proliferation.

In group 2B, significant associations were observed between fibrosis severity and AST levels (R = 0.4; P = 0.04) and between ductular proliferation severity and duration of cholestasis (R = 0.6; P = 0.002) and ERCP-surgery interval (R = 0.6; P = 0.002). After multivariate analysis, both duration of cholestasis (R = 0.5; P = 0.008) and ERCP-surgery interval (R = 0.4; P = 0.01) were independently associated with the severity of ductular proliferation.

Considering the total study population, associations were observed between cholestasis severity and BMI (R = -0.2; P = 0.03), bilirubin levels (R = 0.3; P < 0.001) and ALB (R = -0.2; P = 0.004); between ductular proliferation severity and BMI (R = -0.3; P < 0.001) and ALB levels (R = -0.2; P < 0.001); between the severity of steatosis and age (R = 0.2; P = 0.009); and between the severity of portal inflammation and age (R = 0.2; P = 0.005). After multiple regression analysis, bilirubin levels (R = 0.3; P < 0.001) were independently correlated with cholestasis severity; and BMI (R = -0.2; P = 0.01) was independently correlated with the severity of ductular proliferation. Complete descriptions of correlation coefficients and multiple regression in the different subgroups and in the entire study population are presented in **Table 4**.

**Table 4:** Simple and multiple linear regression analyses between the endpoints considered (severity of histopathological characteristics) and study variables, according to subgroup classification

**Group 1 (no cholestasis)**		

***Endpoint: severity of fibrosis***		

**Variable**	**Correlation coefficient (simple regression)**	**P-value (simple regression)**
Age	0.1	0.2
BMI	0.02	0.8
Bilirubin	0.07	0.5
AST	0.05	0.6
ALT	0.07	0.5
ALP	0.1	0.2
GGT	0.02	0.8
INR	0.05	0.9
Albumin	-0.1	0.2

***Endpoint: severity of cholestasis***				

**Variable**	**Correlation coefficient (simple regression)**	**P-value (simple regression)**	Adjusted coefficient (multiple regression)*	P-value (multiple regression)*
Age	0.2	0.1	NA	NA
BMI	-0.3	*0.01*	-0.2	*0.02*
Bilirubin	-0.06	0.5	NA	NA
AST	-0.06	0.4	NA	NA
ALT	-0.2	0.3	NA	NA
ALP	-0.2	0.06	NA	NA
GGT	0.04	0.4	NA	NA
INR	-0.2	0.1	NA	NA
Albumin	-0.3	*0.01*	-0.2	*0.02*

***Endpoint: severity of ductular proliferation***				

**Variable**	**Correlation coefficient (simple regression)**	**P-value (simple regression)**	Adjusted coefficient (multiple regression)*	P-value (multiple regression)*
Age	0.02	0.7	NA	NA
BMI	-0.3	*0.005*	-0.2	*0.008*
Bilirubin	-0.1	0.2	NA	NA
AST	-0.0001	0.9	NA	NA
ALT	-0.1	0.6	NA	NA
ALP	0.2	0.5	NA	NA
GGT	0.1	0.6	NA	NA
INR	0.04	0.2	NA	NA
Albumin	-0.2	*0.04*	-0.1	0.1

**Group 2 (overall cholestasis)**		

***Endpoint: severity of fibrosis***		

**Variable**	**Correlation coefficient (simple regression)**	**P-value (simple regression)**
Age	0.02	0.9
BMI	0.07	0.6
Duration of cholestasis	0.2	0.1
Bilirubin	0.2	0.2
AST	0.3	*0.06*
ALT	0.08	0.6
ALP	0.4	0.002
GGT	0.1	0.5
INR	0.1	0.3
Albumin	-0.01	0.9

***Endpoint: severity of cholestasis***				

**Variable**	**Correlation coefficient (simple regression)**	**P-value (simple regression)**	Adjusted coefficient (multiple regression)*	P-value (multiple regression)*
Age	0.06	0.7	NA	NA
BMI	0.1	0.5	NA	NA
Duration of cholestasis	0.4	*0.001*	0.3	*0.004*
Bilirubin	0.3	*0.02*	0.2	0.07
AST	0.03	0.8	NA	NA
ALT	0.2	0.2	NA	NA
ALP	0.06	0.7	NA	NA
GGT	0.2	0.1	NA	NA
INR	0.4	*0.01*	0.3	*0.03*
Albumin	-0.1	0.2	NA	NA

***Endpoint: severity of ductular proliferation***		

**Variable**	**Correlation coefficient (simple regression)**	**P-value (simple regression)**
Age	0.07	0.6
BMI	0.2	0.3
Duration of cholestasis	0.6	< *0.001*
Bilirubin	0.06	0.7
AST	0.07	0.6
ALT	0.07	0.6
ALP	0.3	0.06
GGT	0.2	0.09
INR	0.2	0.1
Albumin	0.01	0.8

**Group 2A (active cholestasis)**				

***Endpoint: severity of fibrosis***				

**Variable**	**Correlation coefficient (simple regression)**	**P-value (simple regression)**	Adjusted coefficient (multiple regression)*	P-value (multiple regression)*
Age	0.03	0.2	NA	NA
BMI	-0.04	0.9	NA	NA
Duration of cholestasis	-0.01	0.4	NA	NA
Bilirubin	0.09	0.7	NA	NA
AST	-0.08	0.7	NA	NA
ALT	0.4	*0.03*	0.3	*0.04*
ALP	0.5	*0.01*	0.2	0.08
GGT	0.5	*0.01*	0.5	*0.03*
INR	0.04	0.2	NA	NA
Albumin	-0.1	0.6	NA	NA

***Endpoint: severity of cholestasis***				

**Variable**	**Correlation coefficient (simple regression)**	**P-value (simple regression)**	Adjusted coefficient (multiple regression)*	P-value (multiple regression)*
Age	0.1	0.5	NA	NA
BMI	0.1	0.51	NA	NA
Duration of cholestasis	0.8	*< 0.001*	0.7	*< 0.001*
Bilirubin	0.6	*0.004*	0.5	*0.005*
AST	0.04	0.9	NA	NA
ALT	0.04	*0.9*	NA	NA
ALP	0.08	*0.8*	NA	NA
GGT	0.1	*0.6*	NA	NA
INR	0.3	0.04	0.2	0.1
Albumin	-0.3	< 0.001	-0.2	0.1

***Endpoint: severity of ductular proliferation***				

**Variable**	**Correlation coefficient (simple regression)**	**P-value (simple regression)**	Adjusted coefficient (multiple regression)*	P-value (multiple regression)*
Age	0.1	0.5	NA	NA
BMI	0.1	0.7	NA	NA
Duration of cholestasis	0.7	< *0.001*	0.7	< *0.001*
Bilirubin	0.4	0.08	NA	NA
AST	0.1	0.7	NA	NA
ALT	0.2	0.3	NA	NA
ALP	0.3	0.2	NA	NA
GGT	0.3	0.2	NA	NA
INR	0.7	*0.03*	0.2	0.9
Albumin	-0.7	*< 0.001*	-0.5	0.06

**Group 2B (resolved cholestasis)**		

***Endpoint: severity of fibrosis***		

**Variable**	**Correlation coefficient (simple regression)**	**P-value (simple regression)**
Age	0.5	0.7
BMI	0.01	1.0
ERCP-surgery interval	0.4	0.1
Duration of cholestasis	0.06	0.8
Bilirubin	0.07	0.7
AST	0.4	*0.04*
ALT	0.3	0.1
ALP	0.1	0.6
GGT	0.2	0.3
INR	0.1	0.8
Albumin	-0.04	0.6

***Endpoint: severity of cholestasis***		

**Variable**	**Correlation coefficient (simple regression)**	**P-value (simple regression)**
Age	0.02	0.9
BMI	0.2	0.3
ERCP - surgery interval	-0.1	0.6
Duration of cholestasis	0.3	0.2
Bilirubin	0.2	0.2
AST	0.2	0.3
ALT	0.3	0.2
ALP	0.3	0.1
GGT	0.2	0.3
INR	0.1	0.9
Albumin	0.1	0.7

***Endpoint: severity of ductular proliferation***				

**Variable**	**Correlation coefficient (simple regression)**	**P-value (simple regression)**	Adjusted coefficient (multiple regression)*	P-value (multiple regression)*
Age	0.1	0.7	NA	NA
BMI	0.3	0.1	NA	NA
ERCP - surgery interval	0.6	*0.002*	0.4	*0.01*
Duration of cholestasis	0.6	*0.002*	0.5	*0.008*
Bilirubin	0.01	1.0	NA	NA
AST	0.01	0.9	NA	NA
ALT	0.1	0.7	NA	NA
ALP	0.1	0.7	NA	NA
GGT	0.1	0.6	NA	NA
INR	0.1	0.5	NA	NA
Albumin	-0.1	0.6	NA	NA

**Groups 1 + 2 (entire study population)**		

***Endpoint: severity of fibrosis***		

**Variable**	**Correlation coefficient (simple regression)**	**P-value (simple regression)**
Age	-0.1	0.2
BMI	-0.1	0.4
Bilirubin	-0.04	0.8
AST	-0.1	0.5
ALT	0.01	0.7
ALP	0.2	*0.02*
GGT	0.01	*0.3*
INR	0.02	0.5
Albumin	-0.1	0.07

***Endpoint: severity of cholestasis***				

**Variable**	**Correlation coefficient (simple regression)**	**P-value (simple regression)**	Adjusted coefficient (multiple regression)*	P-value (multiple regression)*
Age	0.1	0.1	NA	NA
BMI	-0.2	0.03	-0.1	0.1
Bilirubin	0.3	< 0.001	0.3	< 0.001
AST	-0.05	0.4	NA	NA
ALT	-0.05	*0.4*	NA	NA
ALP	0.1	*0.4*	NA	NA
GGT	0.02	*0.8*	NA	NA
INR	0.01	0.4	NA	NA
Albumin	-0.2	0.004	0.05	0.9

***Endpoint: severity of ductular proliferation***				

**Variable**	**Correlation coefficient (simple regression)**	**P-value (simple regression)**	**Variable**	**Correlation coefficient (simple regression)**
Age	-0.01	0.9	NA	NA
BMI	-0.3	< 0.001	-0.2	0.001
Bilirubin	0.2	0.04	0.1	0.1
AST	0.03	0.7	NA	NA
ALT	0.1	0.1	NA	NA
ALP	-0.1	0.1	NA	NA
GGT	-0.1	0.2	NA	NA
INR	0.01	*0.3*	NA	NA
Albumin	-0.2	*< 0.001*	-0.03	0.2

***Endpoint: severity of steatosis***		

**Variable**	**Correlation coefficient (simple regression)**	**P-value (simple regression)**
Age	0.2	*0.009*
BMI	0.1	0.2
Bilirubin	-0.04	0.6
AST	0.01	*0.2*
ALT	0.06	0.6
ALP	-0.1	0.2
GGT	-0.04	1.0
INR	-0.002	0.5
Albumin	0.1	0.2

***Endpoint: severity of portal inflammation***		

**Variable**	**Correlation coefficient (simple regression)**	**P-value (simple regression)**
Age	0.2	*0.005*
BMI	0.1	0.06
Bilirubin	0.05	0.4
AST	-0.003	*0.8*
ALT	-0.1	0.5
ALP	0.01	0.7
GGT	-0.02	0.9
INR	0.02	0.8
Albumin	0.1	0.1

BMI = body mass index; AST = aspartate aminotransferase; ALT = alanine aminotransferase; ALP = alkaline phosphatase; GGT = gamma-glutamyl transferase; INR = international normalized ratio; ERCP = endoscopic retrograde cholangiopancreatography; NA = not applicable. *When applicable; Italics indicate statistical significance.

### Liver histology, bilirubin levels and duration of cholestasis

Cholestasis was more frequent in individuals with bilirubin levels over 7 mg/dl (P = 0.04). Other histological features did not differ according to bilirubin levels (**Table 5**).

**Table 5:** Frequencies of liver histological features according to bilirubin levels

	Bilirubin < 3 mg/dl	Bilirubin 3 – 7 mg/dl	Bilirubin > 7 mg/dl	P-value
N	150	15	4	NA
Steatosis, N (%)	60 (40%)	6 (40%)	1 (25%)	0.8
Fibrosis, N (%)	90 (60%)	8 (53.3%)	2 (50%)	0.8
Biliary hepatopathy, N (%)	50 (33.3%)	7 (46.7%)	3 (75%)	0.2
Cholestasis, N (%)	40 (26.7%)	7 (46.7%)	3 (75%)	*0.04*
Cholangitis, N (%)	0	1 (6.7%)	1 (25%)	0.2
Ductular proliferation, N (%)	58 (38.7%)	6 (40%)	2 (50%)	0.9
Portal inflammation, N (%)	47 (31.3%)	6 (40%)	2 (50%)	0.6

N = number of individuals. Italics indicate statistical significance.

Among the individuals who experienced biliary obstructions, there were significantly higher frequencies of cholestasis (P < 0.001), biliary hepatopathy (P < 0.001) and ductular proliferation (P < 0.001) (**Table 6**).

**Table 6:** Frequencies of liver histological features according to duration of cholestasis

	Cholestasis < 15 days	Cholestasis ≥ 15 days	P-value
N	15	39	NA
Steatosis, N (%)	8 (53.3%)	9 (23.1%)	0.05
Fibrosis, N (%)	8 (53.3%)	8 (20.5%)	0.04
Biliary hepatopathy, N (%)	0	35 (89.7%)	< *0.001*
Cholestasis, N (%)	0	23 (59%)	*< 0.001*
Cholangitis, N (%)	0	2 (5.1%)	1.0
Ductular proliferation, N (%)	0	27 (69.2%)	*< 0.001*
Portal inflammation, N (%)	6 (40%)	12 (30.8%)	0.8

N = number of individuals. Italics indicate statistical significance.

## DISCUSSION

The presence of biliary hepatopathy in a population without a clinical history of jaundice may indicate occurrence of subclinical and spontaneously resolved cholestatic events (gallstone migration, for example), but with maintenance of the histopathological alteration indefinitely. Rangaswamy et al. previously observed, among individuals who underwent cholecystectomy and did not present evidence of biliary obstruction, that histopathological signs of acute cholestasis occurred in 9.1% of their sample. Moreover, Patel et al. also observed histopathological cholestasis in 10% of their patients who underwent uncomplicated cholecystolithiasis cholecystectomy.^9^^,^^10^ The possibility of occurrences of resolved subclinical cholestatic events or undetected biliary diseases of undetermined clinical importance was raised in these previous studies, and this is reinforced by the findings of the current study.

The presence of biliary hepatopathy was associated with lower BMI and longer duration of cholestasis in both groups with a history of cholestasis, whether resolved or not. This suggested that some degree of nutritional impairment occurred and that the clinical duration of cholestasis is relevant in relation to histopathological evolution. The impact of extrahepatic cholestasis on nutritional status and weight is related to several pathophysiological changes, among which lipid malabsorption stands out, along with poor appetite and reduced intake in certain contexts.^11^^,^^12^

In individuals with resolved cholestasis who underwent ERCP, a lengthier time elapsed between the procedures, and cholecystectomy was associated with the presence of biliary hepatopathy. There was also a significant correlation between this interval and the severity of ductular proliferation.

Hence, it seems that performing surgery as early as possible may be beneficial. This may be due to the possibility of undetected subclinical cholestatic events during the time out. This finding reinforces the evidence previously described by Jee et al., Mador et al. and Nebiker et al., who demonstrated in different population studies on individuals who had previous episodes of acute biliary pancreatitis that a delay in performing cholecystectomy was associated with higher occurrence of new biliary complications, especially obstruction, during the waiting period for surgery.^13^^,^^14^^,^^15^ Similarly, Moody et al., in a meta-analysis study, observed that early cholecystectomy reduced the frequency of readmissions due to biliary complications, among individuals with previous episodes of acute biliary pancreatitis.^16^

The duration of obstruction was significantly associated with the severity of both cholestasis and ductular proliferation. This highlights the progressive aspect of these changes in relation to the duration of the causative condition and, of course, emphasizes the importance of early treatment in order to avoid the development of chronic disease. Previous studies have shown that the severity of functional and structural changes in the liver is time-dependent, but with varying individual susceptibility. Functional recovery after decompression is not immediate in animal models, with persistent short-term hepatocytic insufficiency.^17^^,^^18^^,^^19^

Steatosis and portal inflammation were significantly associated with age in the general population and did not differ between groups. This suggested that non-alcoholic fatty liver disease (NAFLD) had a greater influence on these changes than did biliary conditions. Older age is a recognized risk factor for occurrence of NAFLD and also for progression to the most deleterious components of its histopathological spectrum, especially steatohepatitis and fibrosis/cirrhosis.^20^^,^^21^

In the study population, treatment and resolution of the obstruction did not lead to full reversal of the histopathological changes associated with cholestasis, in a significant number of patients. There is evidence of mitigation of fibrosis and other liver histological abnormalities later on, after resolution of complete biliary obstructions, but complete resolution does not occur in all affected individuals.^22^-^23^ Olguín et al. demonstrated in a murine model that the time elapsed between the obstruction and its correction is determinant for the possibility of histological reversal.^24^ In addition, the correlation between the observed changes and laboratory tests was not appropriate, thus demonstrating the importance of histopathological examination in these cases.

Based on the results from the present study, it is advisable that any individual with a history of biliary obstruction, even if undergoing treatment and with evidence of resolution in laboratory and imaging examinations, should be accompanied through long-term follow-up. It needs to be borne in mind that the reversal of such biliary obstruction-associated liver changes may not be complete and that long-term changes are possible in this context.

The current study presents limitations that should be taken into consideration. Its retrospective design precludes ultimate conclusions on possible causal connections. Moreover, liver biopsies are subject to sampling error, since histopathological changes may be heterogeneously distributed in the liver parenchyma. Nevertheless, the findings of this study are significant and should be considered within their proper context.

## CONCLUSIONS

Transient cholestasis was associated with significant histopathological changes, even after its resolution. The duration of obstruction correlated with the severity of histopathological changes, especially cholestasis and ductular proliferation. This emphasizes the need for early treatment of obstructive cholestasis.

## References

[1] European Association for the Study of the Liver (2009). EASL Clinical Practice Guidelines: management of cholestatic liver diseases. J Hepatol.

[2] de Vries E, Beuers U. (2017). Management of cholestatic disease in 2017. Liver Int.

[3] Björnsson E, Gustafsson J, Borkman J, Kilander A. (2008). Fate of patients with obstructive jaundice. J Hosp Med.

[4] Zollner G, Trauner M. (2008). Mechanisms of cholestasis. Clin Liver Dis.

[5] Lee RG., Lee RG (1994). Cholestasis and biliary obstruction. Diagnostic Liver Pathology.

[6] Panqueva RPL. (2014). Approaches to Pathological Diagnosis of Cholestatic Diseases. Rev Col Gastroenterol.

[7] Georgiev P, Jochum W, Heinrich S (2008). Characterization of time-related changes after experimental bile duct ligation. Br J Surg.

[8] Crawford AR, Lin XZ, Crawford JM. (1998). The normal adult human liver biopsy: a quantitative reference standard. Hepatology.

[9] Rangaswamy R, Singh CG, Singh HM, Punyabati P, Nyuwi KT. (2017). Impact of Biliary Calculi on the Liver. J Clin Diagn Res.

[10] Patel G, Jain A, Singh M (2018). Liver biopsy in gall stone disease: a prospective study in patients undergoing cholecystectomy. Int Surg J.

[11] Zaina FE, Parolin MB, Lopes RW, Coelho JC. (2004). Prevalence of malnutrition in liver transplant candidates. Transplant Proc.

[12] Rioux KP, Beck PL, Hoppin AG (2000). Differential leptin responses to acute and chronic biliary obstruction in rats. J Hepatol.

[13] Jee SL, Jarmin R, Lim KF, Raman K (2018). Outcomes of early versus delayed cholecystectomy in patients with mild to moderate acute biliary pancreatitis: A randomized prospective study. Asian J Surg.

[14] Mador BD, Panton ON, Hameed SM. (2014). Early versus delayed cholecystectomy following endoscopic sphincterotomy for mild biliary pancreatitis. Surg Endosc.

[15] Nebiker CA, Frey DM, Hamel CT, Oertli D, Kettelhack C. (2009). Early versus delayed cholecystectomy in patients with biliary acute pancreatitis. Surgery.

[16] Moody N, Adiamah A, Yanni F, Gomez D. (2019). Meta-analysis of randomized clinical trials of early versus delayed cholecystectomy for mild gallstone pancreatitis. Br J Surg.

[17] Younes RN, Vydelingum NA, Derooij P (1991). Metabolic alterations in obstructive jaundice: effect of duration of jaundice and bile-duct decompression. HPB Surg.

[18] Bala L, Tripathi P, Choudhuri G, Khetrapal CL. (2011). Restoration of hepatocytes function following decompression therapy in extrahepatic biliary obstructed patients: metabolite profiling of bile by NMR. J Pharm Biomed Anal.

[19] Soares PFDC, Gestic MA, Utrini MP (2019). Epidemiological profile, referral routes and diagnostic accuracy of cases of acute cholangitis among individuals with obstructive jaundice admitted to a tertiary-level university hospital: a cross-sectional study. Sao Paulo Med J.

[20] Younossi Z, Anstee QM, Marietti M (2018). Global burden of NAFLD and NASH: trends, predictions, risk factors and prevention. Nat Rev Gastroenterol Hepatol.

[21] Cazzo E, Jimenez LS, Gestic MA (2018). Type 2 Diabetes Mellitus and Simple Glucose Metabolism Parameters may Reliably Predict Nonalcoholic Fatty Liver Disease Features. Obes Surg.

[22] Sikora SS, Srikanth G, Agrawal V (2008). Liver histology in benign biliary stricture: fibrosis to cirrhosis… and reversal?. J Gastroenterol Hepatol.

[23] Hammel P, Couvelard A, O’Toole D (2001). Regression of liver fibrosis after biliary drainage in patients with chronic pancreatitis and stenosis of the common bile duct. N Engl J Med.

[24] Olguín HJ, Hernández JL, Guzman DC, Medina RA. (2011). Reversibility of hepatic histological damage after surgical temporary obstruction of the common bile duct in a murine model. Int J Biomed Sci.

